# Cross-Species Transmission Risks of a Quail-Origin H7N9 Influenza Virus from China Between Avian and Mammalian Hosts

**DOI:** 10.3390/v17101402

**Published:** 2025-10-21

**Authors:** Cheng Zhang, Yifei Jin, Huan Cui, Zhongyi Wang, Zhaoliang Chen, Lei Zhang, Sihui Song, Bing Lu, Zhendong Guo

**Affiliations:** 1 Changchun Veterinary Research Institute, Chinese Academy of Agricultural Sciences, 573 Tulip Street, Changchun 130122, China; 2 College of Veterinary Medicine, Hebei Agricultural University, 2596 Lucky South Street, Baoding 071000, China; 3 Academy of Military Medical Sciences, Academy of Military Sciences, 20 Dongdajie Road, Beijing 100071, China

**Keywords:** H7N9 avian influenza virus, quail, pathogenicity, cross-species transmission, viral aerosols

## Abstract

The H7N9 influenza viruses, which are capable of causing severe respiratory syndrome in humans, were first discovered to infect humans in 2013 and continue to pose a persistent public health threat. Quail has been proposed as a potential intermediate host that may facilitate the emergence of novel reassorted influenza A viruses with the capacity to infect humans across species barriers; however, information on the biological characterization of quail H7N9 remains limited. In this study, we isolated and identified an avian H7N9 influenza virus from quails, designated as A/quail/Hebei/CH06-07/2018 (H7N9) and abbreviated as CH06-07, in Hebei, China. Phylogenetic analyses revealed that both the HA gene and the NA gene of CH06-07 were clustered in the Eurasian lineage. Furthermore, CH06-07 exhibited binding affinity for both α2,3-linked and α2,6-linked sialic acid receptors and demonstrated high pathogenicity in both quails and mice. Notably, transmission studies revealed that CH06-07 not only exhibited efficient inter-quail transmission and inter-guinea pig transmission but also demonstrated effective cross-species transmission. Importantly, infected quails and guinea pigs generated significant quantities of viral aerosols (≥18,998 ± 1672 copies per liter of air at 3 days post-infection), and infectious viruses were successfully recovered from environmental aerosols. These findings highlight the necessity for continuous surveillance of the prevalence of quail-origin H7N9 influenza A viruses in poultry populations due to their potential threat to human health.

## 1. Introduction

Human infections with H7N9 avian influenza viruses (AIVs) were first reported in China in 2013 [1] and continued to be a public health concern until 2019 [2]. Since then, no new human cases have been documented over the past five years, largely attributable to the implementation of effective prevention strategies, such as poultry vaccination programs and enhanced market interventions [3,4]. However, H7N9 AIVs are still circulating in poultry populations, and remain a potential threat to both the poultry industry and public health [5,6]. Over the period from 2013 to 2017, five waves of human infections with H7N9 AIVs were observed in China [7,8,9]. In the early stage, H7N9 AIVs exhibited low pathogenicity (LP). Since 2016, however, H7N9 AIVs have demonstrated high pathogenicity (HP) following the insertion of multiple amino acid residues at the hemagglutinin (HA) cleavage site [10]. Infection with HP H7N9 AIVs results in a mortality rate of approximately 50% in humans [11,12], whereas the mortality rate among infected poultry approaches 100% [13,14,15]. Consequently, HP H7N9 AIVs represent a significant threat to both human health and the poultry industry.

Influenza viruses can be transmitted through direct contact, indirect contact, or via aerosols [16]. In comparison to non-aerosol-transmitted influenza viruses, aerosol-transmitted influenza viruses are more readily transmissible between hosts and are more likely to trigger an epidemic or pandemic [17,18]. However, the transmission pattern of H7N9 remains to be elucidated. Previous studies have indicated that exposure to infected poultry or contaminated environments in live poultry markets constitutes one of the primary risk factors for H7N9 infection in humans [1,19]. However, approximately 45% of H7N9-infected patients reported no prior contact with poultry [20].

Waterfowl serve as the natural reservoir hosts of influenza A viruses. However, AIVs isolated from waterfowl generally exhibit limited replication capabilities in mammals due to species-specific barriers [21]. For cross-species transmission, AIVs first require host-adaptive genetic mutations to establish basic replication capacity in mammals [22]. Concurrently, an intermediate host that is susceptible to both avian and human influenza viruses, acting as a “mixing vessel”, plays a crucial role in the evolutionary process of influenza. Viral reassortment may occur within this “mixing vessel”, enabling the generation of novel genotypes with enhanced mammalian infectivity and transmissibility [23,24]. Quails exhibit widespread susceptibility to at least 14 influenza subtypes, including both mammalian and avian influenza viruses [25]. Furthermore, quails possess both α2,3-linked and α2,6-linked sialic acid receptors in their trachea, lungs, and intestines [26,27,28]. Therefore, quails can function as effective intermediate hosts for novel reassorted IAVs with pandemic potential [25,29]. Although H7N9 AIVs of quail origin have been investigated in previous studies [30,31,32], there remains limited information regarding the characteristics, cross-species transmission, and exposure risks associated with these viruses.

The mouse model is an excellent experimental model for evaluating host adaptation and pathogenesis of IAVs in mammals [33]. The guinea pig model has been extensively utilized to investigate the transmissible capacity of IAVs due to its susceptibility to influenza infection, its disease progression closely resembling that of humans, and its safety and availability [34,35]. Therefore, in this study, the mouse model was employed to assess the pathogenicity of the quail-origin H7N9 strain in mammals, while the guinea pig model was utilized to evaluate the transmissibility of the quail-origin H7N9 strain in mammals.

In this study, a quail-origin H7N9 strain, designated as A/Quail/Hebei/CH06-07/2018 (H7N9) and abbreviated as CH06-07, was isolated from quail swabs collected in Hebei province in 2018. The quails were sourced from a closed quail farm operated by a large-scale breeding company. The company submitted samples of sick quails for diagnostic testing, and we identified H7N9 AIVs through PCR analysis. Oropharyngeal and cloacal swabs were collected from a total of 50,000 quails before they were culled in accordance with the relevant regulations of the Chinese government. The detection rate of H7N9 was found to be greater than 91%. Phylogenetic analysis, receptor binding specificity, pathogenicity, and transmissibility of the CH06-07 strain were systematically investigated. Additionally, the risk of cross-species transmission between quails and guinea pigs, as well as the environmental viral aerosols generated by the infected animals, was comprehensively assessed. This study highlights the importance of monitoring H7N9 viruses in quails and will help in pandemic preparedness.

## 2. Materials and Methods

### 2.1. Ethics Statement

All the animal studies were performed in strict accordance with the guidelines for animal welfare of the World Organization for Animal Health [36]. The experimental protocols involving animals were approved by the Animal Care and Use Committee of the Changchun Veterinary Research Institute, Chinese Academy of Agricultural Sciences (approval number: SCXK 20210008, date: 4 February 2021). All experiments involving the influenza A (H7N9) virus were performed in an animal biosecurity level 3 laboratory at the Changchun Veterinary Research Institute.

### 2.2. Viruses

The H7N9 virus used in this study was isolated from swabs collected from quails exhibiting influenza-like symptoms at a closed quail farm located in Hebei Province in 2018. The virus was designated as A/quail/Hebei/CH06-07/2018 (H7N9) (hereafter abbreviated as CH06-07; GenBank accession numbers: MT498676 and MT498678), which is an HP strain. The viruses were subsequently propagated in 9-day-old specific pathogen-free chicken eggs (Merial Vital Laboratory Animal Technology Company, Beijing, China) and stored at −80 °C.

### 2.3. Phylogenetic and Sequence Analysis

Viral RNA extraction and PCR amplification were performed using the methods described in a previous study [37]. The PCR products were purified and sequenced by Comate Bioscience Company Limited (Changchun, Jilin, China). All sequence data were assembled and analyzed using the SEQMAN program (DNASTAR, Madison, WI, USA). Reference sequences utilized in the present analysis (as detailed in Supplemental Table S1) were retrieved from the National Center for Biotechnology Information (NCBI) GenBank database through nucleotide BLAST searches (available at http://www.ncbi.nlm.nih.gov/BLAST, accessed on 21 May 2021). These reference sequences were selected based on their high nucleotide identity to the sequences obtained in this study as indicated by BLAST results, ensuring representativeness of closely related viral strains. Sequences were aligned using Cluster W. Phylogenetic analysis was subsequently conducted using the distance-based neighbor-joining (NJ) algorithm implemented in MEGA 7 software, with 1000 bootstrap replicates to evaluate the robustness of the inferred phylogeny.

### 2.4. Receptor-Binding Specificity Assay

The receptor-binding assays were performed according to the methodology detailed in our previous study [38]. We included two strains of IVs with known receptor-binding properties as controls. The avian-origin A/chicken/Hebei/HB777/2006 (H5N1) virus exhibited preferential binding to α2,3–linked sialic acid (SA) receptors, whereas the human isolate A/California/04/2009 (H1N1) demonstrated recognition of α2,6–linked SA receptors. The receptor-binding specificities of the H7N9 viruses were determined by HA assays conducted using 1% chicken red blood cells (cRBCs) and sheep red blood cells (sRBCs). For sialidase treatment, 90 μL of a 10% cRBC suspension was incubated with 10 μL of α2,3-sialidase (50 mU/μL; TaKaRa, Dalian, China) for 10 min at 37 °C. The sample was subsequently washed twice with PBS, followed by centrifugation at 1500 rpm for 5 min after each wash. It was then resuspended in PBS to a final working concentration of 1% and stored at 4 °C. For *Vibrio cholerae* neuraminidase (VCNA; Roche, San Francisco, CA, USA) treatment, 90 μL of a 10% cRBC suspension was incubated with 10 μL of VCNA (50 mU/μL) for 1 h at 37 °C. The sample was subsequently washed twice with PBS, followed by centrifugation at 1500 rpm for 5 min after each wash. It was then resuspended in PBS to achieve a final working concentration of 1% and stored at 4 °C. For the HA assay, viruses were serially diluted twofold with 50 µL of PBS and mixed with 50 µL of a 1% RBC suspension in a 96-well plate. HA titers were determined following incubation at room temperature for 20 min.

Receptor affinity was also determined using a solid-phase direct virus binding assay, as described previously [39]. In brief, the influenza virus was purified using a sucrose density gradient ultracentrifugation method and titrated to approximately 2.0 × 105 hemagglutination units per mL. The purified virus was gently labeled with Alexa 555, after which the HA titer was re-evaluated. The labeled virus was subsequently allowed to bind to 80 N-glycans spotted on N-hydroxysuccinimide-derivatized slides, as described previously [40]. Following the cleaning and drying of the chips, the data were scanned and acquired using an InnoScan 1100 aluminum fluorescence imager (Innopsys, Carbonne, France).

### 2.5. Animals

A total of 48 unvaccinated female Japanese quails aged 3 weeks (*Coturnix japonica*) (Hongda Experimental Animal Company, Changchun, China), 35 female BALB/c mice aged six weeks (Merial Vital Laboratory Animal Technology Company, Beijing, China), and 18 female Hartley strain guinea pigs aged six weeks (Merial Vital Laboratory Animal Technology Company, Beijing, China) were used in this study. Details regarding their usage are outlined in the experimental design. Animals were maintained under strictly controlled environmental conditions, with the temperature set at 24 ± 2 °C, relative humidity ranging from 40% to 70%, and a 12 h light/dark cycle.

### 2.6. Monitoring the Survival Rate of Quails

A total of 10 quails were randomly allocated into two groups (n = 5): a control group and a challenged group. These groups were used for the observation of clinical symptoms and survival testing. The challenged group was intranasally inoculated with 100 µL of the CH06-07 solution containing 10^6.0^ EID_50_ viruses (50 µL per nostril), while the control group was intranasally inoculated with an equal volume of PBS. The survival of the quails was monitored daily for 14 days post-challenge, and quails that lost more than 25% of their initial body weight were humanely euthanized. The method of euthanasia for quail is cervical dislocation under deep anesthesia. The quails in each group were housed separately to prevent cross-contamination.

### 2.7. Tissue Distribution of H7N9 in Quails

A total of 20 quails were randomly allocated into two groups: the control group (n = 5) and the challenged group (n = 15). These groups were used to evaluate the tissue distribution of the virus and the pathological characteristics of the lungs. At 48 h post-infection (hpi), 3 quails were humanely euthanized to analyze the tissue distribution of the virus. Nine tissues (heart, liver, spleen, lung, kidney, brain, cecum, pancreas, and trachea) were collected from the infected quails for the determination of virus titers. Briefly, the tissues were weighed, and 0.1 g of each tissue sample was placed in 1 mL of PBS containing 100 U/mL penicillin, resulting in a 10% weight/volume tissue homogenate. The tissue samples were homogenized using a Tissue Lyser (QIAGEN, Hilden, Germany) and subsequently centrifuged at 10,000 rpm for 10 min. The resulting supernatant was carefully harvested and serially diluted 10-fold in sterile PBS. Thereafter, the diluted samples were inoculated into 9-day-old embryonated eggs via the allantoic cavity route. Following incubation at 37 °C for 48 to 72 h, HA activity was tested, and the EID_50_ was determined using the Reed and Muench method. The lungs from both the uninfected quails and the infected quails euthanized at 48 hpi were fixed with formalin, embedded in paraffin, and subsequently stained with hematoxylin and eosin for pathological examination.

### 2.8. Monitoring of Body Weight Changes and the Survival Rate of Mice

A total of 10 BALB/c mice were randomly allocated into two groups (n = 5): the control group and the challenged group. The challenged group was anesthetized with isoflurane and subsequently inoculated intranasally with 50 µL of the CH06-07 solution containing 10^6.0^ EID_50_ viruses, while the control group was inoculated intranasally with an equal volume of PBS. The body weight and survival of the mice were monitored daily for 14 days post-challenge, and mice that lost more than 25% of their initial body weight were humanely euthanized.

### 2.9. Tissue Distribution of H7N9 in Mice

A total of 25 BALB/c mice were randomly allocated into two groups: the control group (n = 5) and the challenged group (n = 20). These groups were used to evaluate the tissue distribution of the virus and the pathological characteristics of the lungs. The challenged group was anesthetized with isoflurane and subsequently inoculated intranasally with 50 µL of the CH06-07 solution containing 10^6.0^ EID_50_ viruses, whereas the control group was inoculated intranasally with an equal volume of PBS. At 3, 5 and 7 days post-infection (dpi), 3 mice were euthanized. Six tissues, including heart, liver, spleen, lungs, kidneys, and brain, were collected from the infected mice for the determination of virus titers. Briefly, the preparation method for the tissue homogenates and subsequent viral titration was the same as that used for the quail tissues. The lungs from both the uninfected mice and the infected mice euthanized at 3 dpi were fixed with formalin, embedded in paraffin, and subsequently stained with hematoxylin and eosin for pathological examination.

### 2.10. Transmissibility of CH06-07 Between Different Animals and Environmental Aerosol Collection

To evaluate the transmissibility of CH06-07 between animals via direct contact and aerosol transmission, three donor animals were inoculated with 10^6.0^ EID_50_ of CH06-07. At 24 hpi, the three donor animals were transferred to a clean cage and co-housed with 3 naive recipient animals for direct contact transmission studies. Additionally, another three naive recipient animals were housed in a wire-frame cage adjacent to the donor animals for aerosol transmission studies. The distance between the infected and aerosol-exposed animals was 5 cm. Therefore, a ratio of 3:3:3 for donors, direct-contact recipients, and aerosol-contact recipients was employed in the study. The donor animals and recipient animals were either quails or guinea pigs, leading to a total of four distinct transmission experiment groups: assessment of viral transmissibility from quails to quails, guinea pigs to guinea pigs, quails to guinea pigs, and guinea pig to quails, respectively.

The specimens were collected while the animals were under anesthesia. For swab collection, oropharyngeal and cloacal swabs were collected from quails at 1, 3, and 5 days post-inoculation or exposure and subsequently soaked in 1 mL of PBS. For nasal wash solution collection, 200 μL of PBS containing 1% penicillin-streptomycin was administered at 1, 3, 5, and 7 days post-inoculation or exposure to collect nasal washes from each guinea pig. The specimens were stored at −80 °C prior to viral titration.

Viral titers in nasal washes and swabs were quantified by titration using 9-day-old SPF embryonated chicken eggs, thereby assessing the extent of viral shedding [41]. The seroconversion of animals was not evaluated in this study. During the experimental process, the CH06-07 virus demonstrated high transmissibility. In this case, it is conventional to prioritize the assessment of infectious virus shedding in infected and exposed animals as a direct indicator of transmission, considering its substantial biological significance. However, for viruses with limited transmission capacity, seroconversion testing functions as an indirect indicator of transmission.

A liquid sampler (All Glass Impinger 30, AGI-30; ACE Glass Inc., Vineland, NJ, USA) combined with a sampling pump (Smart air microbial sampler-ZR-2000, Zhongrui Intelligent Instrument Co., Ltd., Qingdao, China) was used to collect the biological particles in the air. The sampling flow rate was 12.5 L/min, and the pressure drop across the orifice was 41 Hg/cm. The sampling port was placed between the inoculated animals and the airborne contact animals in the transmission cage. The sampling solution was PBS containing 1% penicillin-streptomycin and the volume was 30 mL. The sampling time was 1 h, and the AGI-30 sampler was placed on ice during the hour of collection. Air sampling was conducted while both donor and recipient animals were housed in their cages and prior to the animals being anesthetized for specimen collection. After the air collection period was complete, the liquid collected by the AGI-30 sampler was enriched with 0.5% chicken red blood cells and incubated at 4 °C for 1 h. After the hemagglutination of the influenza virus, the whole mixture was centrifuged at 1500 rpm for 5 min. The supernatant was discarded, and the chicken red blood cells were resuspended in 1 mL of PBS. Half of the resuspended sample was subsequently titrated into 9-day-old embryonated eggs, and the EID_50_ was determined using the Reed and Muench method. Viral RNA was extracted from the other half of the samples using TRIzol reagent (Thermo Fisher Scientific Inc., Waltham, MA, USA), and the viral RNA copy numbers were subsequently quantified by quantitative RT-PCR (primers (5′→3′): F-CGC AGA AGG GGC TCT TGG ACT A; R-ATT CTC ATG CCT GAT TAG TGG GTT G). Based on the data obtained from viral titer determination and quantitative RT-PCR analysis, we evaluated the changes in viral aerosol concentration within the airborne transmission assessment device during the experiment.

### 2.11. Statistical Analysis

One-way analysis of variance (ANOVA), performed using GraphPad Prism 9.5.1 software (San Diego, CA, USA), was employed to analyze the differences between different groups. *p* < 0.05 was considered statistically significant. Pearson correlation coefficient analysis was used to examine the correlation between the viral concentration in the air and the degree of viral shedding in inoculated animals. All the assays were performed in triplicate and are representative of at least 3 independent experiments. The error bars represent the standard deviations.

## 3. Results

### 3.1. Phylogenetic Analysis of the Surface Genes of the Quail-Origin H7N9 Virus CH06-07

**Figure 1:**
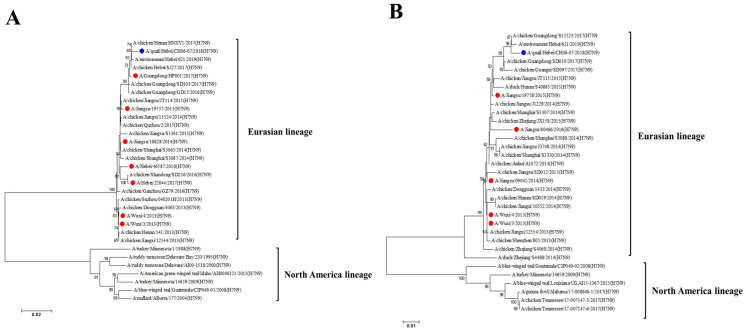
Phylogenetic analysis of the HA and NA genes. The distance-based neighbor joining method in MEGA7 software was used to construct a phylogenetic tree of the HA (**A**) and NA (**B**) genes. The reliability of the trees was assessed via a bootstrap analysis. The horizontal distance is proportional to the genetic distance. Influenza virus isolates in this study are marked with solid blue circles. Human H7N9 viruses are marked with solid red circles.

The full-length sequences of the influenza virus A/quail/Hebei/CH06-07/2018 (H7N9) (abbreviated as CH06-07) were compared to those of known influenza viruses deposited in the GenBank database. Six of the eight gene segments of CH06-07 (PB1, PA, HA, NA, M, and NS) exhibited the highest degree of nucleotide sequence homology with the chicken strains isolated in 2017 (Table 1). In contrast, the PB2 and NP segments demonstrated the highest degree of nucleotide sequence homology with A/duck/Japan/AQ-HE29-22/2017(H7N9) (Table 1). The CH06-07 virus possesses several amino acid sites that have been reported to be associated with increased pathogenicity and transmissibility (Table 2). The HA and NA genes of the CH06-07 virus are classified within the Eurasian lineage (Figure 1). The HA gene exhibits the closest evolutionary genetic relationship with A/chicken/Henan/HNXY1/2017(H7N9), while the NA gene is closely related to A/environment/Hebei/621/2019(H7N9). The HA and NA genes exhibit close genetic and evolutionary relationships with chicken strains isolated in recent years.

### 3.2. The Quail-Origin H7N9 Virus Ch06-07 Exhibited Binding Affinity for Both Avian and Human Sialic Acid Receptors

**Figure 2:**
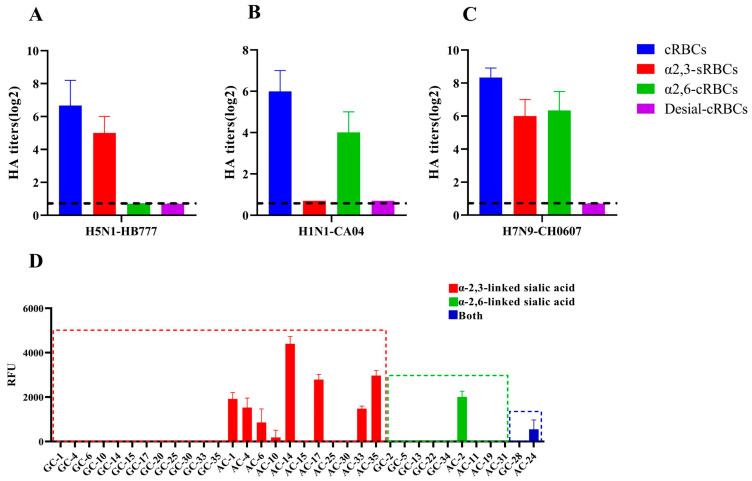
Receptor binding specificity of the quail-origin H7N9 (CH06-07) virus. (**A**–**C**) Agglutination activity (hemagglutinin [HA] titers) of the quail H7N9 virus in red blood cells (RBCs) from different species. cRBCs are chicken red blood cells (with α2,3-linked sialic acid receptors and α2,6-linked sialic acid receptors). α2,3-sRBCs refer to sheep red blood cells (with only α2,3-linked sialic acid receptors). α2,6-cRBCs are chicken red blood cells treated with α2,3-sialidase (with only α2,6-linked sialic acid receptors). Desial-cRBCs refer to chicken red blood cells treated with VCNA (no receptors). The values are shown as the means and standard deviations of three independent experiments. (**D**) Receptor binding specificity analyzed by a glycan microarray analysis. The values on the X-axis represent the number of N-Glycolylneuraminic acid (Neu5Gc) glycan and N-acetylneuraminic acid (Neu5Ac) glycan, respectively. “GC” refers to Neu5Gc and “AC” refers to Neu5Ac—both are critical members of sialic acid. Specifically, Neu5Ac is the most widely distributed and representative form of sialic acid, serving as the main type in mammalian tissues; Neu5Gc is generally present in non-neural tissues and body fluids of most deuterostomes, including echinoderms and vertebrates [54]. Notably, Neu5Ac is the primary sialic acid form in the human body [55]. The Neu5Gc and Neu5Ac glycan ID and structure of the specific glycan used in the glycan microarray analysis are detailed in Supplemental Table S2. The vertical bars denote the fluorescence binding signal intensity.

The key determinant of the cross-species transmission of influenza A virus is its receptor binding specificity [52,53]. Consequently, we assessed the receptor binding specificity of the CH06-07 virus using four distinct types of red blood cells (RBCs). The cRBCs (chicken RBCs) contain both α2,3-linked and α2,6-linked sialic acid receptors. The α2,3-sRBCs (sheep RBCs) contain exclusively α2,3-linked sialic acid receptors. The α2,6-cRBCs (cRBCs treated with α2,3-sialidase) contain exclusively α2,6-linked sialic acid receptors. The desial-cRBCs (cRBCs treated with VCNA) contain no sialic acid receptors. The HA titers are shown in Figure 2. These results indicate that the CH06-07 virus exhibits affinity for both avian-like (α2,3-linked) receptors and human-like (α2,6-linked) receptors, suggesting that CH06-07 not only possesses the ability to infect poultry but also harbors the potential to infect mammals. We further investigated the glycan-binding profiles of the CH06-07 virus using an isoform microarray. Consistent with the results of the HA assays, the isoform microarray analysis revealed that the CH06-07 virus displayed affinity for glycans containing branched α2,3-linked sialic acids and α2,6-linked sialic acids (Figure 2D).

### 3.3. The Quail-Origin H7N9 Virus Ch06-07 Exhibited High Pathogenicity in Quails

Five quails in each group were intranasally inoculated with 10^6.0^ EID_50_ of CH06-07 (H7N9) or PBS. All quails in the infection group developed clinical signs. At 48 hpi, fibrinosuppurative nasal secretions were observed in the infected quails.

**Figure 3:**
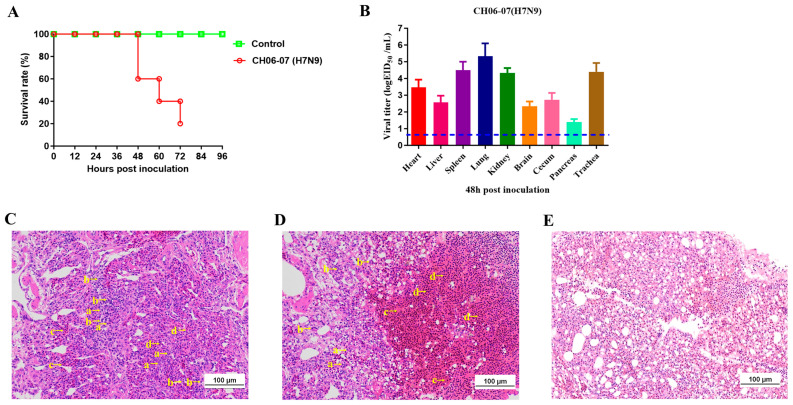
Pathogenicity of CH06-07 (H7N9) in quails. Five quails per group were intranasally inoculated with 10^6.0^ EID_50_ of CH06-07 (H7N9) or PBS. (**A**) Percent survival of the infected quails. In the infection group, two quails (2/5) succumbed to infection within 48 hpi, one (1/5) died within 60 hpi, one (1/5) died within 72 hpi, and the remaining quail (1/5) was humanely euthanized at 72 hpi due to a body weight loss exceeding 25% of its initial weight (predefined humane endpoints). In contrast, all quails in the control group remained healthy throughout the study period. (**B**) Tissues were collected from infected quails at 48 hpi (n = 3), and virus titers were determined in 9-day-old specific pathogen-free embryonated eggs. (**C**–**E**) Histopathological analysis of the lungs of quails inoculated with CH06-07 (**C**,**D**) or PBS (**E**). At 48 hpi, lung tissues were collected from the quails, fixed with formalin, embedded in paraffin, and stained with hematoxylin and eosin. The images were obtained at a magnification of ×20. (Arrow a) Lymphocyte infiltration, (arrow b) macrophage infiltration, (arrow c) congestion of the lung wall, and (arrow d) congestion of the pulmonary capillary wall. The scale bar represents 100 µm.

In the infection group, two quails (2/5) succumbed to infection within 48 hpi, one (1/5) died within 60 hpi, one (1/5) died within 72 hpi, and the remaining quail (1/5) was humanely euthanized at 72 hpi due to a body weight loss exceeding 25% of its initial weight. This humane euthanasia procedure was performed when quail exhibited severe clinical signs—such as persistent weight loss, inability to eat or drink, and severe respiratory distress—that met the predefined humane endpoints, aiming to avoid unnecessary pain and suffering of the animals. This practice is a critical component of ensuring animal welfare and is widely adopted in similar virus infection studies [56,57,58,59,60,61]. The natural mortality rate of the infected quails was 80% (4/5). In contrast, all quails in the control group remained healthy throughout the study period (Figure 3A). Tissues from the infected quails were collected at 48 hpi and titrated in 9-day-old embryonated chicken eggs. The CH06-07 virus was detected in all collected tissues, including the heart, liver, spleen, lungs, kidneys, brain, cecum, pancreas, and trachea. The lung tissue exhibited the highest viral titer (10^5.33±0.76^ EID_50_/mL; ANOVA analysis: *p* < 0.05 compared with the viral titer in spleen, kidney or trachea), while the pancreas demonstrated the lowest viral titer (10^1.40±0.17^ EID_50_/mL; ANOVA analysis: *p* < 0.05 compared with the viral titer in brain or cecum) (Figure 3B). In addition, we performed a histopathological analysis of the collected lungs, and the lungs of the infected quails exhibited lymphocyte infiltration, macrophage infiltration, congestion of the lung wall and congestion of the pulmonary capillary wall (Figure 3C,D). In contrast, no obvious pathological damage was observed in the lung tissues of the control group (Figure 3E). The histological analysis revealed severe histopathological changes in quails infected with the CH06-07 virus.

### 3.4. The Quail-Origin H7N9 Virus Ch06-07 Exhibited High Pathogenicity in Mice

**Figure 4:**
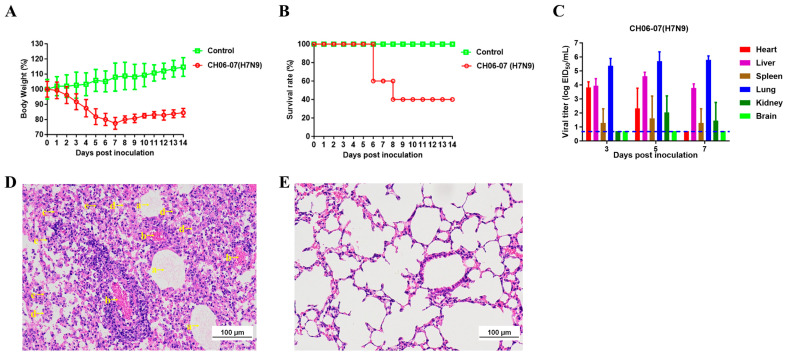
Pathogenicity of CH06-07 (H7N9) in mice. Five mice per group were intranasally inoculated with 10^6.0^ EID_50_ of the CH06-07 virus or with PBS. (**A**) Mouse body weights were monitored daily for 14 days. The values are presented as the average percentages of overall body weight loss with respect to the initial body weights ± standard deviations (SDs). (**B**) Percent survival of the infected mice. (**C**) Tissues were collected from infected mice at 3, 5 and 7 dpi (n = 3), and virus titers were determined in 9-day-old specific pathogen-free embryonated eggs. (**D**,**E**) Histopathological analysis of the lungs of mice inoculated with CH06-07 (**D**) or PBS (**E**). At 3 dpi, lung tissues were collected from the mice, fixed with formalin, embedded in paraffin, and stained with hematoxylin and eosin. The images were obtained at a magnification of × 20. (Arrow a) Cellulose exudation, (arrow b) lung congestion, (arrow c) focal bleeding, (arrow d) lymphocyte infiltration, and (arrow e) macrophage infiltration. The scale bar represents 100 µm.

Five six-week-old female BALB/c mice were anesthetized with isoflurane and intranasally inoculated with a 10^6.0^ EID_50_ solution of CH06-07 to evaluate the pathogenicity of the CH06-07 virus in a mammalian host. After 2 dpi, the mice developed clinical manifestations, including an unkempt coat, loss of appetite, and varying degrees of weight loss. Mice infected with the CH06-07 virus experienced rapid weight loss and demonstrated a mortality rate of 60% (Figure 4A,B). In contrast, mice in the control group exhibited normal behavior throughout the study, and their body weights gradually increased.

Next, we quantified the viral titer of CH06-07 in six tissues (heart, liver, spleen, lungs, kidneys, and brain) collected from the infected mice (Figure 4C). The virus was detectable in the heart, liver, lungs, spleen and kidneys, with the highest virus titers observed in the lungs. At 3 dpi, the viral titer in the lungs was 10^5.37±0.52^ EID_50_/mL; at 5 dpi, it was 10^5.70±0.66^ EID_50_/mL; and at 7 dpi, it was 10^5.78±0.29^ EID_50_/mL. Infectious viruses were not detected in the brains of mice, but were present in the brains of quails (Figure 3B). Given that the H7N9 virus was originally isolated from quails, it may possess inherent host-specific adaptations favoring replication in this avian species. These adaptations are reflected in more efficient viral replication and higher pathogenicity in quails compared to mice—a distinction further supported by the respective lethality rates (80% in quails versus 60% in mice). We propose that host adaptation-driven differences in viral replication efficiency and pathogenicity contribute to the observed disparity in viral replication within the brain.

In addition, we performed a histopathological analysis of the collected lungs. The lungs of the infected mice exhibited fibrinous exudation, congestion, focal hemorrhage, lymphocytic infiltration, and macrophage infiltration (Figure 4D). In contrast, no obvious pathological damage was observed in the lung tissues of the control group (Figure 4E). The histological analysis demonstrated that the mice infected with the CH06-07 virus developed severe histopathological changes.

In conclusion, based on the findings from mouse model studies, we deduced that the CH06-07 virus displays pronounced pathogenicity in mammals without requiring prior adaptation to mammalian hosts.

### 3.5. The Quail-Origin H7N9 Virus Demonstrated Cross-Species Transmission Between Quail and Guinea Pigs, and Infectious Viral Particles Were Successfully Recovered from Environmental Aerosols

**Figure 5:**
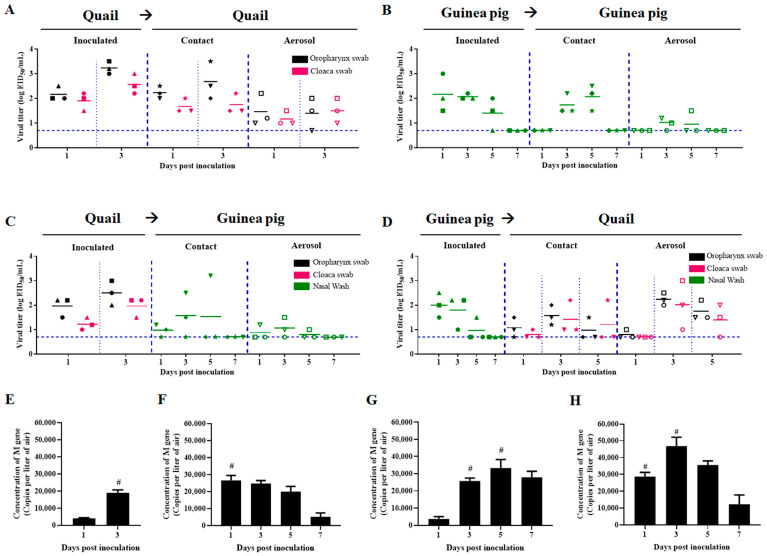
Transmissibility of CH06-07 (H7N9) in quails and guinea pigs and viral aerosols generated during aerosol transmission. Both contact transmission and aerosol transmission experiments were conducted. In each experiment, the species of recipient animals was identical for both the contact and aerosol transmission setups. (**A**) Contact and aerosol transmissibility of CH06-07 (H7N9) in quails. (**B**) Contact and aerosol transmissibility of CH06-07 (H7N9) in guinea pigs. (**C**) Contact and aerosol transmissibility of CH06-07 (H7N9) from quails to guinea pigs. (**D**) Contact and aerosol transmissibility of CH06-07 (H7N9) from guinea pigs to quails. Each point represents the virus titer of a sample from an individual animal. The mean virus titer of each group is shown as a short horizontal line. The horizontal dashed lines indicate the lower limit of virus detection. (**E**) Concentration of viral aerosols during transmission among quails. (**F**) Concentration of viral aerosols during transmission among guinea pigs. (**G**) Concentration of viral aerosols during transmission from quails to guinea pigs. (**H**) Concentration of viral aerosols during transmission from guinea pigs to quails. # indicates that the infectious virus was recovered from the aerosol samples.

Nasal washes or swabs were collected every other day from all animals for the detection of viral shedding. In the H7N9 virus transmission experiment between quails (Figure 5A), the virus was detected in all cloacal and oropharyngeal swabs collected from recipient quails at 1 dpi. The direct contact and aerosol transmission efficiency among quails was 100%. In the H7N9 virus transmission experiment between guinea pigs (Figure 5B), at 3 dpi, the virus was detected in the nasal washes of 3 guinea pigs in the direct contact group and 2 guinea pigs in the aerosol transmission group. Consequently, the direct contact transmission efficiency among guinea pigs was 100%, and the aerosol transmission efficiency was 66.7%. In the H7N9 virus transmission experiment from quails to guinea pigs (Figure 5C), at 3 dpi, the virus was detected in the nasal washes from two guinea pigs in the direct contact group and two guinea pigs in the aerosol transmission group. The transmission efficiency of direct contact from quails to guinea pigs was 66.7%, and the efficiency of aerosol transmission from quails to guinea pigs was also 66.7%. In the H7N9 virus transmission experiment from guinea pigs to quails (Figure 5D), at 3 dpi, the virus was detected in cloacal and oropharyngeal swabs from all recipient quails. The transmission efficiency of both direct contact and aerosol transmission from guinea pigs to quails was 100%. In conclusion, we demonstrated that the CH06-07 virus exhibited efficient transmission capabilities in both quails and guinea pigs and efficiently transmitted across species, either from quails to guinea pigs or vice versa (Figure 5A–D), thereby highlighting the pandemic potential of the quail-origin H7N9 virus.

Environmental aerosols were collected during the transmission experiments, and half of the samples were titrated using 9-day-old embryonated eggs. The recovery of infectious viral particles was more feasible at 1 dpi and 3 dpi, potentially contributing to aerosol transmission (Table 3). The other half of the samples were analyzed by Q-PCR to determine the viral copy number (Figure 5E–H). A comparison of the data in Figure 5A and Figure 5E, as well as those in Figure 5B,F, revealed a strong positive correlation between the viral concentration in the air and the degree of viral shedding in inoculated animals (Pearson Correlation Coefficient Analysis: *r* = 1, viral titers in oropharynx swabs [Figure 5A] versus viral concentrations in the air [Figure 5E]; *r* = 0.97, viral titers in nasal washes [Figure 5B] versus viral concentrations in the air [Figure 5F]). In the transmission experiments among quails, the concentration of viral aerosols increased over time and reached a peak at 3 dpi, with a concentration of 18,998 ± 1672 copies per liter of air (Figure 5E). In contrast, during the transmission experiments among guinea pigs, the concentration of viral aerosols peaked at 1 dpi, with a concentration of 26,586 ± 2865 copies per liter of air (Figure 5F), and subsequently decreased gradually over time. In the transmission experiments from quails to guinea pigs, the concentration of viral aerosols initially increased and subsequently decreased, peaking at 5 dpi with a concentration of 33,257 ± 4986 copies per liter of air (Figure 5G). In the transmission experiments from guinea pigs to quails, the concentration of viral aerosols also initially increased and subsequently decreased, peaking at 3 dpi with a concentration of 46,852 ± 5253 copies per liter of air (Figure 5H).

## 4. Discussion

Influenza A viruses undergo continuous evolution, and the exchange of entire gene fragments via reassortment is a key driver in the emergence of novel epidemic, pandemic and zoonotic strains [62,63]. China offers an ideal environment for the emergence and circulation of novel influenza viruses with the potential for cross-species transmission [64]. The coexistence of extensive poultry farming, poly-culture farming practices, and the live poultry market system collectively shapes a distinctive influenza ecosystem [65].

From 2013 to 2017, the LPAI H7N9 influenza virus caused five waves of human infection epidemic [12]. In December 2016, during the fifth epidemic wave, the first case of human infection with HPAI H7N9 virus, characterized by a multi-basic cleavage site motif, was reported. The emergence of HPAI H7N9 viruses may pose an increased threat to human health, as the multi-basic cleavage site in the HA protein is associated with enhanced pathogenicity in mammals [66,67]. The quail-origin H7N9 in this study also possesses a multi-basic cleavage site in the HA protein, indicating its classification as an HPAI strain and highlighting its significant risk to both poultry and humans. The new risk assessment tools also indicated that, in comparison to H5N1, H7N9 exhibits a higher potential for pandemic risk due to its greater capacity for mammalian adaptation and possible human-to-human transmission [44,45].

Previous studies have indicated that chickens serve as a critical host species for H7N9 viruses in live poultry markets [17]. Nevertheless, compared with chickens, influenza viruses originating from quails may present a higher risk of transmission, potentially leading to more severe public health threats [30]. A previous experimental transmission study suggested that quails could be infected with H7N9 at a relatively low infectious dose and achieve relatively high infection rates. Quails might represent a critical species in the infection and transmission dynamics of this virus within live animal markets [32]. Additionally, quails could function as amplifying hosts, releasing substantial quantities of viable H7N9 viruses into the environment, thereby posing a potential threat to human health in shared environment [46]. However, comprehensive investigations into the biological characteristics of quail-origin H7N9 viruses remain limited.

In this study, we isolated a quail-origin H7N9 virus in Hebei, China, designated as CH06-07. Genetic evolutionary analysis revealed that the HA and NA genes of the CH06-07 virus originated from the Eurasian lineage [4]. The HA gene exhibits the closest genetic evolutionary relationship with A/chicken/Henan/HNXY1/2017(H7N9), while the NA gene shows a close genetic association with A/environment/Hebei/621/2019(H7N9). Recently, the Eurasian lineage of the H7N9 influenza virus has become the predominant epidemic-causing lineage in China, in contrast to the North American lineage. Since the late 1990s, a diverse range of poultry influenza subtypes/genotypes, including H5, H9, H6, H7 and H10 (hosted by species such as chickens, ducks, and quails), have been circulating in China [68,69,70,71]. These subtypes/genotypes contribute to a rich gene pool that facilitates further inter- or intra-subtype reassortments [72]. Furthermore, the prevalence of these viruses in China, their capacity to infect humans, and their potential to reassort with human H3N2 and/or H1N1 viruses in the future raise concerns regarding their pandemic potential [73,74]. Our research emphasizes the critical need for continuous monitoring of the evolutionary dynamics of the H7N9 virus and for expediting the evaluation of the pathogenicity and transmission capacity of newly isolated viruses.

The sequence analysis revealed that CH06-07 possesses several amino acid residues previously identified as being associated with enhanced pathogenicity and increased transmission potential (Table 2): one in the HA subunit (186V), two in the PB2 subunit (292I, 526R), one in the PB1 subunit (368V), two in the PA subunit (356R, 409N), two in the M1 subunit (30D, 156D), and one in the NS1 subunit (42S). The HA 186V site enables the virus to acquire the capability of binding to human-like receptors, and it may facilitate the virus in undergoing amino acid mutations, thereby obtaining the ability to transmit infections across species [75,76,77]. Receptor binding properties are currently among the critical determinants that influence the capacity of viruses to infect different species. AIVs tend to exhibit a preference for avian-like receptors. When AIVs cross the species barrier to infect mammals, they typically undergo evolutionary changes in receptor binding preferences, shifting from an exclusive affinity for avian-like receptors to a dual affinity for both avian and human-like receptors [78,79,80]. The CH06-07 virus exhibits two receptor-binding properties, which enable it to infect both avian species and mammals. The PB2 subunit of the previously isolated human H7N9 influenza virus harbors a 526R site, which has been demonstrated to enhance the pathogenicity of the virus in mice [43]. Similarly, the CH06-07 virus also contains a 526R site in its PB2 subunit, potentially contributing to increased pathogenicity. Additionally, the 356R and 409N sites in the PA subunit, as well as the 30D site in the M1 subunit of the CH06-07 virus, may also play a role in enhancing pathogenicity in mice [48,49,50]. The NS1 subunit of the CH06-07 virus contains a 42S site, which significantly augments the interferon antagonistic potency upon viral infection in mice [51]. Consequently, the therapeutic efficacy of interferon is partially compromised, thereby contributing to increased viral pathogenicity in mice. Moreover, the 292I site in the PB2 subunit, the 368V site in the PB1 subunit, and the 156D site in the M1 subunit of the CH06-07 virus have been reported to be associated with enhanced transmissibility in mammals [42,47,50], and the 292I site also facilitates the replication and dissemination of influenza viruses among avian species [42].

Chicken-origin H7N9 viruses have been well documented as important zoonotic pathogens [66,81,82,83,84]. Compared with chicken-origin H7N9 viruses, the quail-origin H7N9 strain (CH06-07) in this study demonstrated similar receptor binding specificity—affinity for both avian and human sialic acid receptors [66]—along with comparable tissue tropism and high pathogenicity in avian hosts [66,81,82]. However, CH06-07 exhibited greater pathogenicity and transmissibility in mammals, thereby posing a significant threat to public health and agriculture. While different chicken-origin H7N9 strains vary in virulence in mice [66,81,82,84], CH06-07 displays lethality in the upper moderate range among reported HAPI chicken-origin H7N9 isolates in mouse models. In contrast to chicken-origin H7N9 viruses, which primarily transmit through direct contact, with limited airborne transmissibility [66,83], CH06-07 demonstrated efficient airborne transmission in both quails and guinea pigs and showed capacity for cross-species transmission (Figure 5).

According to previous studies, the H5N1 reassortant virus containing the 2009/H1N1 viral gene can be transmitted via respiratory droplets in a guinea pig model [85]. Furthermore, H5N6 and H7N9 viruses are capable of being transmitted through direct contact in the same animal model [86,87]. Several research groups have evaluated the airborne transmissibility of H7N9 viruses in a guinea pig model, revealing that certain H7N9 viruses are indeed disseminated among guinea pigs via aerosols [88,89]. In addition, prior investigations have demonstrated that the H7N9 virus can be effectively transmitted between ferrets through both direct contact and aerosols, thereby highlighting potential biosafety concerns [17,90,91]. Our research revealed that the CH06-07 virus exhibits not only efficient transmissibility in quails and guinea pigs but also, notably, the capacity for cross-species transmission, from quails to guinea pigs and vice versa. Moreover, during the transmission experiment, significant levels of viral aerosols were detected, and infectious viral particles were recovered at specific time points. Although the viral aerosols originated from both inoculated and recipient animals which were shedding virus on different schedules, thereby limiting our ability to infer detailed information from the relative timing or overall levels of detected aerosols, this does not alter the warning regarding the potential public health risks associated with the quail-origin H7N9 influenza virus. People who regularly come into contact with quails should take precautions and receive personal protection.

This study has three main limitations: First, the investigation was based solely on a single quail-derived H7N9 strain (CH06-07) and did not include other quail-derived H7N9 strains isolated from different geographic regions or time periods, which may affect the generalizability of the findings. Second, the replication capacity of the virus in human respiratory epithelial cells was not assessed, limiting the ability to directly reflect the potential risk of human infection. Third, although the sample sizes in the animal experiments were sufficient to detect statistically significant differences, they were relatively small.

## 5. Conclusions

Overall, the quail-origin H7N9 virus exhibited typical characteristics associated with highly pathogenic H7N9 viruses. Most of its properties are shared by previously reported strains of highly pathogenic H7N9 viruses [67]. As an important zoonotic pathogen, the quail-origin H7N9 virus poses not only a potential threat to the global livestock and poultry industry’s economy but also a considerable risk to public health. Effective measures must be implemented to control the source of infection. Consequently, given the confirmed risk of aerosol transmission associated with the quail-origin H7N9 virus, it is recommended to install aerosol monitoring systems in quail farms and conduct regular measurements of airborne viral load to enable early detection of aerosol-mediated viral spread and prevent potential pandemics. Additionally, considering the virus’s high pathogenicity in quails, routine pathogen screening of quail flocks should be strengthened to facilitate early elimination of the infection source. Such targeted surveillance and control measures are expected to effectively mitigate the risk of cross-species transmission of the quail-origin H7N9 virus.

## Figures and Tables

**Table 1:** Analysis of nucleotide homology of the respective gene segment of the CH06-07 virus.

Gene	Strain with the Highest Homology	Identity	GenBank Accession Number
PB2	A/duck/Japan/AQ-HE29-22/2017(H7N9)	99.13%	LC315928.1
PB1	A/chicken/Hebei/SD156/2017(H7N9)	99.43%	MH209417.1
PA	A/chicken/Heinan/ZZ01/2017(H7N9)	99.63%	MF319559.1
HA	A/chicken/Heinan/ZZ01/2017(H7N9)	99.24%	MF319554.1
NP	A/duck/Japan/AQ-HE29-22/2017(H7N9)	99.47%	LC315924.1
NA	A/chicken/Guangxi/97/2017(H7N9)	99.57%	MK453330.1
M	A/chicken/Henan/SD163/2017(H7N9)	99.29%	MH209430.1
NS	A/chicken/Heinan/ZZ01/2017(H7N9)	99.64%	MF319558.1

**Table 2:** Specific amino acid sites possessed by CH06-07 virus that are related to enhanced pathogenicity and transmissibility.

Viral Protein	Position	Amino Acid	Comments
PB2	292	I	Increased pathogenicity and transmissibility [42]
	526	R	Increased virulence in mammals (present in some human H7N9 isolates) [43]
HA	186	V	Increased viral binding affinity to human-type receptors [44,45,46]
PB1	368	V	Increased transmission among mammals (This mutation site has been increasingly identified in H7N9 virus strains isolated recently) [47]
PA	356	R	Increased virulence in mice (most avian influenza viruses encode 356R) [48]
	409	N	Increased virulence in mice [49]
M1	30	D	Increased virulence in mice [50]
	156	D	Increased transmissibility in mammals [50]
NS1	42	S	Increased virulence in mice (most avian influenza A viruses encode 42S) [51]

**Table 3:** Viral titer of infectious virus in collected aerosol samples (log EID_50_/mL).

Transmission Direction	Days Post-Infection (dpi)			
	1	3	5	7
quail→quail	ND ^1^	1.03 ± 0.14	ND	ND
guinea pig→guinea pig	1.37 ± 0.52	ND	ND	ND
quail→guinea pig	ND	1.78 ± 0.52	1.12 ± 0.14	ND
guinea pig→quail	1.20 ± 0.25	1.45 ± 0.43	ND	ND

^1^ ND, not detected. The limit of detection of the virus titer was 0.7 (log EID_50_/mL). The recovery rate of infectious viruses collected with the AGI-30 sampler in cages was 91%.

## Data Availability

The data will be made available upon request.

## Supplementary Materials

The following supporting information can be downloaded at: https://www.mdpi.com/article/10.3390/v17101402/s1, Table S1: Basic information of reference strains; Table S2: The Neu5Gc glycan ID and structure of the specific glycan used in the glycan microarray analysis.
