# Supplementary material for: Cross-Species Transmission Risks of a Quail-Origin H7N9 Influenza Virus from China Between Avian and Mammalian Hosts
Source: Viruses. 2025 Oct 21;17(10):1402. doi: 10.3390/v17101402 (PMC12567846; doi:10.3390/v17101402)
Supplement: Supplementary file 1 [file viruses-17-01402-s001.zip › Supplement Table S2.pdf]

Supplement Table 2. The Neu5Gc glycan ID and structure of the specific glycan used in the glycan microarray analysis

No.	Neu5Gc Glycan ID	Structure	No.	Neu5Ac Glycans ID	Structure
GC-1	N002G	Neu5Gcα2-3Galβ1-4GlcNAcβ1-2Manα1-6(Neu5Gcα2-3Galβ1-4GlcNAcβ1-2Manα1-3)Manβ1-4GlcNAcβ1-4GlcNAc-	AC-1	N002	Neu5Acα2-3Galβ1-4GlcNAcβ1-2Manα1-6(Neu5Acα2-3Galβ1-4GlcNAcβ1-2Manα1-3)Manβ1-4GlcNAcβ1-4GlcNAc-
GC-2	N003G	Neu5Gcα2-6Galβ1-4GlcNAcβ1-2Manα1-6(Neu5Gcα2-6Galβ1-4GlcNAcβ1-2Manα1-3)Manβ1-4GlcNAcβ1-4GlcNAc-	AC-2	N003	Neu5Acα2-6Galβ1-4GlcNAcβ1-2Manα1-6(Neu5Acα2-6Galβ1-4GlcNAcβ1-2Manα1-3)Manβ1-4GlcNAcβ1-4GlcNAc-
GC-3	N005G	Neu5Gcα2-3Galβ1-4(Fucα1-3)GlcNAcβ1-2Manα1-6(Neu5Gcα2-3Galβ1-4(Fucα1-3)GlcNAcβ1-2Manα1-3)Manβ1-4GlcNAcβ1-4GlcNAc-	AC-3	N005	Neu5Acα2-3Galβ1-4(Fucα1-3)GlcNAcβ1-2Manα1-6(Neu5Acα2-3Galβ1-4(Fucα1-3)GlcNAcβ1-2Manα1-3)Manβ1-4GlcNAcβ1-4GlcNAc-
GC-4	N012G	Manα1-6(Manα1-3)Manα1-6(Neu5Gcα2-3Galβ1-4GlcNAcβ1-2Manα1-3)Manβ1-4GlcNAcβ1-4GlcNAc-	AC-4	N012	Manα1-6(Manα1-3)Manα1-6(Neu5Acα2-3Galβ1-4GlcNAcβ1-2Manα1-3)Manβ1-4GlcNAcβ1-4GlcNAc-
GC-5	N013G	Manα1-6(Manα1-3)Manα1-6(Neu5Gcα2-6Galβ1-4GlcNAcβ1-2Manα1-3)Manβ1-4GlcNAcβ1-4GlcNAc-	AC-5	N013	Manα1-6(Manα1-3)Manα1-6(Neu5Acα2-6Galβ1-4GlcNAcβ1-2Manα1-3)Manβ1-4GlcNAcβ1-4GlcNAc-
GC-6	N015G	Manα1-6(Manα1-3)Manα1-6(Neu5Gcα2-3Galβ1-4(Fucα1-3)GlcNAcβ1-2Manα1-3)Manβ1-4GlcNAcβ1-4GlcNAc-	AC-6	N015	Manα1-6(Manα1-3)Manα1-6(Neu5Acα2-3Galβ1-4(Fucα1-3)GlcNAcβ1-2Manα1-3)Manβ1-4GlcNAcβ1-4GlcNAc-
GC-7	N022G	Neu5Gcα2-3Galβ1-4GlcNAcβ1-2Manα1-3Manβ1-4GlcNAcβ1-4GlcNAc-	AC-7	N022	Neu5Acα2-3Galβ1-4GlcNAcβ1-2Manα1-3Manβ1-4GlcNAcβ1-4GlcNAc-
GC-8	N023G	Neu5Gcα2-6Galβ1-4GlcNAcβ1-2Manα1-3Manβ1-4GlcNAcβ1-4GlcNAc-	AC-8	N023	Neu5Acα2-6Galβ1-4GlcNAcβ1-2Manα1-3Manβ1-4GlcNAcβ1-4GlcNAc-
GC-9	N025G	Neu5Gcα2-3Galβ1-4(Fucα1-3)GlcNAcβ1-2Manα1-3Manβ1-4GlcNAcβ1-4GlcNAc-	AC-9	N025	Neu5Acα2-3Galβ1-4(Fucα1-3)GlcNAcβ1-2Manα1-3Manβ1-4GlcNAcβ1-4GlcNAc-
GC-10	N032G	Manα1-6(Neu5Gcα2-3Galβ1-4GlcNAcβ1-2Manα1-3)Manβ1-4GlcNAcβ1-4GlcNAc-	AC-10	N032	Manα1-6(Neu5Acα2-3Galβ1-4GlcNAcβ1-2Manα1-3)Manβ1-4GlcNAcβ1-4GlcNAc-
GC-11	N033G	Manα1-6(Neu5Gcα2-6Galβ1-4GlcNAcβ1-2Manα1-3)Manβ1-4GlcNAcβ1-4GlcNAc-	AC-11	N033	Manα1-6(Neu5Acα2-6Galβ1-4GlcNAcβ1-2Manα1-3)Manβ1-4GlcNAcβ1-4GlcNAc-
GC-12	N042G	Neu5Gcα2-3Galβ1-4GlcNAcβ1-2Manα1-6Manβ1-4GlcNAcβ1-4GlcNAc-	AC-12	N042	Neu5Acα2-3Galβ1-4GlcNAcβ1-2Manα1-6Manβ1-4GlcNAcβ1-4GlcNAc-
GC-13	N043G	Neu5Gcα2-6Galβ1-4GlcNAcβ1-2Manα1-6Manβ1-4GlcNAcβ1-4GlcNAc-	AC-13	N043	Neu5Acα2-6Galβ1-4GlcNAcβ1-2Manα1-6Manβ1-4GlcNAcβ1-4GlcNAc-
GC-14	N045G	Neu5Gcα2-3Galβ1-4(Fucα1-3)GlcNAcβ1-2Manα1-6Manβ1-4GlcNAcβ1-4GlcNAc-	AC-14	N045	Neu5Acα2-3Galβ1-4(Fucα1-3)GlcNAcβ1-2Manα1-6Manβ1-4GlcNAcβ1-4GlcNAc-
GC-15	N052G	Neu5Gcα2-3Galβ1-4GlcNAcβ1-2Manα1-6(Manα1-3)Manβ1-4GlcNAcβ1-4GlcNAc-	AC-15	N052	Neu5Acα2-3Galβ1-4GlcNAcβ1-2Manα1-6(Manα1-3)Manβ1-4GlcNAcβ1-4GlcNAc-
GC-16	N053G	Neu5Gcα2-6Galβ1-4GlcNAcβ1-2Manα1-6(Manα1-3)Manβ1-4GlcNAcβ1-4GlcNAc-	AC-16	N053	Neu5Acα2-6Galβ1-4GlcNAcβ1-2Manα1-6(Manα1-3)Manβ1-4GlcNAcβ1-4GlcNAc-
GC-17	N055G	Neu5Gcα2-3Galβ1-4(Fucα1-3)GlcNAcβ1-2Manα1-6(Manα1-3)Manβ1-4GlcNAcβ1-4GlcNAc-	AC-17	N055	Neu5Acα2-3Galβ1-4(Fucα1-3)GlcNAcβ1-2Manα1-6(Manα1-3)Manβ1-4GlcNAcβ1-4GlcNAc-
GC-18	N112G	GlcNAcβ1-2Manα1-6(Neu5Gcα2-3Galβ1-4GlcNAcβ1-2Manα1-3)Manβ1-4GlcNAcβ1-4GlcNAc-	AC-18	N112	GlcNAcβ1-2Manα1-6(Neu5Acα2-3Galβ1-4GlcNAcβ1-2Manα1-3)Manβ1-4GlcNAcβ1-4GlcNAc-
GC-19	N113G	GlcNAcβ1-2Manα1-6(Neu5Gcα2-6Galβ1-4GlcNAcβ1-2Manα1-3)Manβ1-4GlcNAcβ1-4GlcNAc-	AC-19	N113	GlcNAcβ1-2Manα1-6(Neu5Acα2-6Galβ1-4GlcNAcβ1-2Manα1-3)Manβ1-4GlcNAcβ1-4GlcNAc-
GC-20	N115G	GlcNAcβ1-2Manα1-6(Neu5Gcα2-3Galβ1-4(Fucα1-3)GlcNAcβ1-2Manα1-3)Manβ1-4GlcNAcβ1-4GlcNAc-	AC-20	N115	GlcNAcβ1-2Manα1-6(Neu5Acα2-3Galβ1-4(Fucα1-3)GlcNAcβ1-2Manα1-3)Manβ1-4GlcNAcβ1-4GlcNAc-
GC-21	N122G	Galβ1-4GlcNAcβ1-2Manα1-6(Neu5Gcα2-3Galβ1-4GlcNAcβ1-2Manα1-3)Manβ1-4GlcNAcβ1-4GlcNAc-	AC-21	N122	Galβ1-4GlcNAcβ1-2Manα1-6(Neu5Acα2-3Galβ1-4GlcNAcβ1-2Manα1-3)Manβ1-4GlcNAcβ1-4GlcNAc-
GC-22	N123G	Galβ1-4GlcNAcβ1-2Manα1-6(Neu5Gcα2-6Galβ1-4GlcNAcβ1-2Manα1-3)Manβ1-4GlcNAcβ1-4GlcNAc-	AC-22	N123	Galβ1-4GlcNAcβ1-2Manα1-6(Neu5Acα2-6Galβ1-4GlcNAcβ1-2Manα1-3)Manβ1-4GlcNAcβ1-4GlcNAc-
GC-23	N125G	Galβ1-4GlcNAcβ1-2Manα1-6(Neu5Gcα2-3Galβ1-4(Fucα1-3)GlcNAcβ1-2Manα1-3)Manβ1-4GlcNAcβ1-4GlcNAc-	AC-23	N125	Galβ1-4GlcNAcβ1-2Manα1-6(Neu5Acα2-3Galβ1-4(Fucα1-3)GlcNAcβ1-2Manα1-3)Manβ1-4GlcNAcβ1-4GlcNAc-
GC-24	N133G	Neu5Gcα2-3Galβ1-4GlcNAcβ1-2Manα1-6(Neu5Gcα2-6Galβ1-4GlcNAcβ1-2Manα1-3)Manβ1-4GlcNAcβ1-4GlcNAc-	AC-24	N133	Neu5Acα2-3Galβ1-4GlcNAcβ1-2Manα1-6(Neu5Acα2-6Galβ1-4GlcNAcβ1-2Manα1-3)Manβ1-4GlcNAcβ1-4GlcNAc-
GC-25	N134G	Neu5Gcα2-3Galβ1-4GlcNAcβ1-2Manα1-6(Galβ1-4(Fucα1-3)GlcNAcβ1-2Manα1-3)Manβ1-4GlcNAcβ1-4GlcNAc-	AC-25	N134	Neu5Acα2-3Galβ1-4GlcNAcβ1-2Manα1-6(Galβ1-4(Fucα1-3)GlcNAcβ1-2Manα1-3)Manβ1-4GlcNAcβ1-4GlcNAc-
GC-26	N135G	Neu5Gcα2-3Galβ1-4GlcNAcβ1-2Manα1-6(Neu5Gcα2-3Galβ1-4(Fucα1-3)GlcNAcβ1-2Manα1-3)Manβ1-4GlcNAcβ1-4GlcNAc-	AC-26	N135	Neu5Acα2-3Galβ1-4GlcNAcβ1-2Manα1-6(Neu5Acα2-3Galβ1-4(Fucα1-3)GlcNAcβ1-2Manα1-3)Manβ1-4GlcNAcβ1-4GlcNAc-
GC-27	N144G	Neu5Gcα2-6Galβ1-4GlcNAcβ1-2Manα1-6(Galβ1-4(Fucα1-3)GlcNAcβ1-2Manα1-3)Manβ1-4GlcNAcβ1-4GlcNAc-	AC-27	N144	Neu5Acα2-6Galβ1-4GlcNAcβ1-2Manα1-6(Galβ1-4(Fucα1-3)GlcNAcβ1-2Manα1-3)Manβ1-4GlcNAcβ1-4GlcNAc-
GC-28	N145G	Neu5Gcα2-6Galβ1-4GlcNAcβ1-2Manα1-6(Neu5Gcα2-3Galβ1-4(Fucα1-3)GlcNAcβ1-2Manα1-3)Manβ1-4GlcNAcβ1-4GlcNAc-			
GC-29	N155G	Galβ1-4(Fucα1-3)GlcNAcβ1-2Manα1-6(Neu5Gcα2-3Galβ1-4(Fucα1-3)GlcNAcβ1-2Manα1-3)Manβ1-4GlcNAcβ1-4GlcNAc-	AC-29	N155	Galβ1-4(Fucα1-3)GlcNAcβ1-2Manα1-6(Neu5Acα2-3Galβ1-4(Fucα1-3)GlcNAcβ1-2Manα1-3)Manβ1-4GlcNAcβ1-4GlcNAc-
GC-30	N212G	Neu5Gcα2-3Galβ1-4GlcNAcβ1-2Manα1-6(GlcNAcβ1-2Manα1-3)Manβ1-4GlcNAcβ1-4GlcNAc-	AC-30	N212	Neu5Acα2-3Galβ1-4GlcNAcβ1-2Manα1-6(GlcNAcβ1-2Manα1-3)Manβ1-4GlcNAcβ1-4GlcNAc-
GC-31	N213G	Neu5Gcα2-6Galβ1-4GlcNAcβ1-2Manα1-6(GlcNAcβ1-2Manα1-3)Manβ1-4GlcNAcβ1-4GlcNAc-	AC-31	N213	Neu5Acα2-6Galβ1-4GlcNAcβ1-2Manα1-6(GlcNAcβ1-2Manα1-3)Manβ1-4GlcNAcβ1-4GlcNAc-
GC-32	N215G	Neu5Gcα2-3Galβ1-4(Fucα1-3)GlcNAcβ1-2Manα1-6(GlcNAcβ1-2Manα1-3)Manβ1-4GlcNAcβ1-4GlcNAc-	AC-32	N215	Neu5Acα2-3Galβ1-4(Fucα1-3)GlcNAcβ1-2Manα1-6(GlcNAcβ1-2Manα1-3)Manβ1-4GlcNAcβ1-4GlcNAc-
GC-33	N222G	Neu5Gcα2-3Galβ1-4GlcNAcβ1-2Manα1-6(Galβ1-4GlcNAcβ1-2Manα1-3)Manβ1-4GlcNAcβ1-4GlcNAc-	AC-33	N222	Neu5Acα2-3Galβ1-4GlcNAcβ1-2Manα1-6(Galβ1-4GlcNAcβ1-2Manα1-3)Manβ1-4GlcNAcβ1-4GlcNAc-
GC-34	N223G	Neu5Gcα2-6Galβ1-4GlcNAcβ1-2Manα1-6(Galβ1-4GlcNAcβ1-2Manα1-3)Manβ1-4GlcNAcβ1-4GlcNAc-	AC-34	N223	Neu5Acα2-6Galβ1-4GlcNAcβ1-2Manα1-6(Galβ1-4GlcNAcβ1-2Manα1-3)Manβ1-4GlcNAcβ1-4GlcNAc-
GC-35	N225G	Neu5Gcα2-3Galβ1-4(Fucα1-3)GlcNAcβ1-2Manα1-6(Galβ1-4GlcNAcβ1-2Manα1-3)Manβ1-4GlcNAcβ1-4GlcNAc-	AC-35	N225	Neu5Acα2-3Galβ1-4(Fucα1-3)GlcNAcβ1-2Manα1-6(Galβ1-4GlcNAcβ1-2Manα1-3)Manβ1-4GlcNAcβ1-4GlcNAc-
GC-36	N233G	Neu5Gcα2-6Galβ1-4GlcNAcβ1-2Manα1-6(Neu5Gcα2-3Galβ1-4GlcNAcβ1-2Manα1-3)Manβ1-4GlcNAcβ1-4GlcNAc-	AC-36	N233	Neu5Acα2-6Galβ1-4GlcNAcβ1-2Manα1-6(Neu5Acα2-3Galβ1-4GlcNAcβ1-2Manα1-3)Manβ1-4GlcNAcβ1-4GlcNAc-
GC-37	N235G	Neu5Gcα2-3Galβ1-4(Fucα1-3)GlcNAcβ1-2Manα1-6(Neu5Gcα2-3Galβ1-4GlcNAcβ1-2Manα1-3)Manβ1-4GlcNAcβ1-4GlcNAc-			
GC-38	N245G	Neu5Gcα2-3Galβ1-4(Fucα1-3)GlcNAcβ1-2Manα1-6(Neu5Gcα2-6Galβ1-4GlcNAcβ1-2Manα1-3)Manβ1-4GlcNAcβ1-4GlcNAc-			
GC-39	N255G	Neu5Gcα2-3Galβ1-4(Fucα1-3)GlcNAcβ1-2Manα1-6(Galβ1-4(Fucα1-3)GlcNAcβ1-2Manα1-3)Manβ1-4GlcNAcβ1-4GlcNAc-	AC-39	N255	Neu5Acα2-3Galβ1-4(Fucα1-3)GlcNAcβ1-2Manα1-6(Galβ1-4(Fucα1-3)GlcNAcβ1-2Manα1-3)Manβ1-4GlcNAcβ1-4GlcNAc-
GC-40	N003G1	Neu5Acα2-6Galβ1-4GlcNAcβ1-2Manα1-6(Neu5Gcα2-6Galβ1-4GlcNAcβ1-2Manα1-3)Manβ1-4GlcNAcβ1-4GlcNAc-			
GC-41	N003G2	Neu5Gcα2-6Galβ1-4GlcNAcβ1-2Manα1-6(Neu5Acα2-6Galβ1-4GlcNAcβ1-2Manα1-3)Manβ1-4GlcNAcβ1-4GlcNAc-			
