# Supplementary material for: Cross-Species Transmission Risks of a Quail-Origin H7N9 Influenza Virus from China Between Avian and Mammalian Hosts
Source: Viruses. 2025 Oct 21;17(10):1402. doi: 10.3390/v17101402 (PMC12567846; doi:10.3390/v17101402)
Supplement: Supplementary file 1 [file viruses-17-01402-s001.zip › Supplement Table S1.pdf]

Supplementary Table 1. Basic information of reference strains

Serial number	Strain	Host	Source	Separation time	HA gene accession number	NA gene accession number
1	A/chicken/Shaanxi/SD183/2017 (H7N9)	chicken	China	2017	MH209443.1	MH209445.1
2	A/duck/Guangxi/S21445/2017 (H7N9)	duck	China	2017	MH209499.1	MH209501.1
3	A/chicken/Heilongjiang/ BQC01/2017(H7N9)	chicken	China	2017	MG298777.1	MG298781.1
4	A/chicken/Inner Mongolia/SD186/2017(H7N9)	chicken	China	2017	MH209451.1	MH209453.1
5	A/duck/Japan/AQ-HE29-52/2017(H7N9)	duck	Japan	2017	LC374952.1	LC374953.1
6	A/chicken/Tibet/S3268/2017 (H7N9)	chicken	China	2017	MH209331.1	MH209333.1
7	A/chicken/Yunnan/SD210/2017 (H7N9)	chicken	China	2017	MH209483.1	MH209485.1
8	A/chicken/Anhui/SD206/2017 (H7N9)	chicken	China	2017	MH209475.1	MH209477.1
9	A/environment/Fujian/SD160/ 2017(H7N9)	-	China	2017	MH209563.1	MH209565.1
10	A/Guangdong/HP001/2017 (H7N9)	human	China	2017	KY643843.1	KY643847.1
11	A/chicken/Guangdong/GD15/ 2016(H7N9)	chicken	China	2016	KY751058.1	KY751124.1
12	A/environment/Guangdong/S12412/2017(H7N9)	-	China	2017	MH209539.1	MH209541.1
13	A/chicken/Guangdong/SD008/2017(H7N9)	chicken	China	2017	MF630037.1	MF630039.1

14	A/Kunming/KMCDC-YHY/2017(H7N9)	human	China	2017	MG366901.1	MG366903.1
15	A/pigeon/Wuxi/0405007G/2013 (H7N9)	pigeon	China	2013	KM879381.1	KM879383.1
16	A/Hong Kong/470129/2013(H7N9)	human	China	2013	KF952508.1	KF952507.1
17	A/chicken/Suzhou/040201H/ 2013(H7N9)	chicken	China	2013	KM879365.1	KM879367.1
18	A/duck/Mongolia/128/2008 (H7N9)	duck	Mongolia	2008	AB829332.1	
19	A/bean goose/Korea/SH20-17/2008 (H7N3)	bean goose	South Korea	2008	KC609768.1	KC609795.1
20	A/Anas crecca/Spain/1460/2008 (H7N9)	Anas crecca	Spain	2008	HQ244407.1	HQ244409.1
21	A/duck/Bangladesh/26992/2015 (H7N9)	duck	Banglades h	2015	KY635780.1	KY635541.1
22	A/goose/Czech Republic/1848-K9/2009(H7N9)	goose	Czech Republic	2009	GU060482.1	GU060484.1
23	A/wild duck/Korea/MHC39-26/2011(H7N9)	wild duck	South Korea	2011	KC609780.1	KC609806.1
24	A/turkey/Minnesota/1/1988(H7N9)	turkey	USA	1988	CY014786.1	CY014788.1
25	A/ruddy turnstone/Delaware Bay/220/1995(H7N9)	ruddy turnstone	USA	1995	CY127253.1	CY127255.1
26	A/ruddy turnstone/DE/1538/2000(H7N9)	ruddy turnstone	USA	2000	CY186010.1	CY186012.1
27	A/blue-winged teal/Guatemala/ CIP049-01/2008(H7N9)	blue-winged teal	Guatemal	2008	CY067670.1	CY067672.1
28	A/mallard/Minnesota/AI09-3770/2009(H7N9)	mallard	USA	2009	CY186002.1	CY186004.1

29	A/mallard/Alberta/177/2004 (H7N9)	mallard	USA	2004	KX827377.1	KX827373.1
30	A/northern shoverl/Mississippi/11OS145/2011(H7N9)	northern shoveler	USA	2011	CY133649.1	CY133651.1
31	A/blue-winged teal/Louisiana/UGAI15-1367/2015(H7N9)	blue-winged teal	USA	2015	KY013764.1	KY013766.1
32	A/blue-winged teal/Louisiana/UGAI15-1692/2015(H7N9)	blue-winged teal	USA	2015	KY013772.1	KY013774.1
